# Exploring the Use of Artificial Intelligence-Enhanced Virtual Reality in Teaching and Evaluating Communication Skills in Urology: A Pilot Study

**DOI:** 10.7759/cureus.109258

**Published:** 2026-05-20

**Authors:** Mohammed Saad, Aleece Warner, Alfred Brunt, Shahid Khan

**Affiliations:** 1 Urology, Barts Health NHS Trust, London, GBR

**Keywords:** artificial intelligence in healthcare, comunication, teaching technology, urology research, virtual reality simulation

## Abstract

Introduction: Effective communication is a core competency in healthcare, yet traditional training methods often face limitations in realism, scalability, and learner engagement. This study evaluated the perceptions of healthcare professionals regarding the use of artificial intelligence (AI)-enhanced virtual reality (VR) simulations for communication skills training in urology.

Methods: A total of 45 healthcare professionals (N = 45) participated in a haematuria role-play simulation using AI-enhanced VR via Bodyswaps© software. Participants completed pre- and post-simulation questionnaires using a standard five-point Likert scale to measure perceptions of the potential of AI-VR to improve communication skills, usefulness, agreement with AI-generated feedback, realism compared to clinical practice, and overall enjoyability. Although Likert-scale responses are ordinal, they were treated as approximately interval data for statistical analysis. Descriptive statistics and one-sample t-tests against the neutral midpoint (3.0) were conducted to assess whether participant perceptions differed significantly from neutrality, with significance set at α = 0.05.

Results: Prior to the simulation, participants (n = 23, 51.11%) reported belief in the potential of AI-VR to improve communication skills by responding above the neutral midpoint (M = 3.844 ± 0.9034). Post-simulation, participants (n = 40, 88.89%) showed increased perceptions of usefulness (M = 4.636 ± 0.6851). High levels of agreement with AI-generated feedback were reported by participants (n = 35, 77.78%) (M = 4.2 ± 0.9195), and participants (n = 40, 88.89%) rated the simulation as useful (M = 4.5 ± 0.6949). Regarding realism, participants (n = 34, 73.33%) rated the simulation positively (M = 4.159 ± 0.9135), and participants (n = 41, 91.11%) found the experience enjoyable (M = 4.465 ± 0.8549). All results reached statistical significance (p < 0.0001).

Conclusion: The findings suggest that AI-enhanced VR simulations have potential as effective and engaging tools for communication skills training in urology. The positive responses across all measured domains support further integration of immersive AI technologies into clinical education.

## Introduction

Effective communication, which includes clear verbal expression, active listening, empathy, and non-verbal signals, is essential to patient care. These components directly influence patient trust, adherence to treatment plans, and overall safety. Approaches such as the teach-back method have improved patient understanding in chronic disease management, leading to a reduction in hospital readmissions [1,2]. Additionally, non-verbal communication through gestures, facial expressions, and posture strengthens connections and reinforces key medical messages [3,4].

Emerging technologies such as virtual reality (VR) and artificial intelligence (AI) are rapidly transforming various industries, including healthcare, with implications for medical education. Traditional communication training methods often struggle with challenges related to realism, scalability, and engagement, especially in a world increasingly influenced by social media and technological advancements. In this context, VR offers immersive learning environments that promote empathy and facilitate comprehensive assessment and communication [5,6].

AI-powered virtual patients facilitate scalable and realistic simulations, providing immediate and structured feedback on both clinical knowledge and communication [7]. These AI systems support unlimited practice opportunities and personalised adaptive learning paths, all of which contribute to improved communication skills. Students have also reported positive feedback regarding the realism and cognitive engagement of these systems [8]. Additionally, AI-enhanced VR simulations are valuable in community-based teaching, where traditional resources are limited [9]. Scoping reviews have highlighted that AI/VR applications range from surgical risk communication to inter-professional counselling, although challenges related to AI responsiveness and technology adoption still persist [10].

In the UK, VR-based initiatives have led to increased learner confidence, better recognition of emotional and non-verbal cues, and enhanced readiness for challenging clinical interactions [11-13]. The incorporation of AI-driven avatars and VR environments has been shown to promote positive learning outcomes through repeated practice in safe, controlled settings. Despite the potential of these technologies, challenges related to AI responsiveness and the adoption of new technologies remain [10].

This study aimed to evaluate the effectiveness of AI-based VR simulations in teaching and assessing urology communication skills, with a particular focus on healthcare professionals' perceptions of using these innovative tools to enhance communication competency.

## Materials and methods

Creating the VR role play

**Figure 1:**
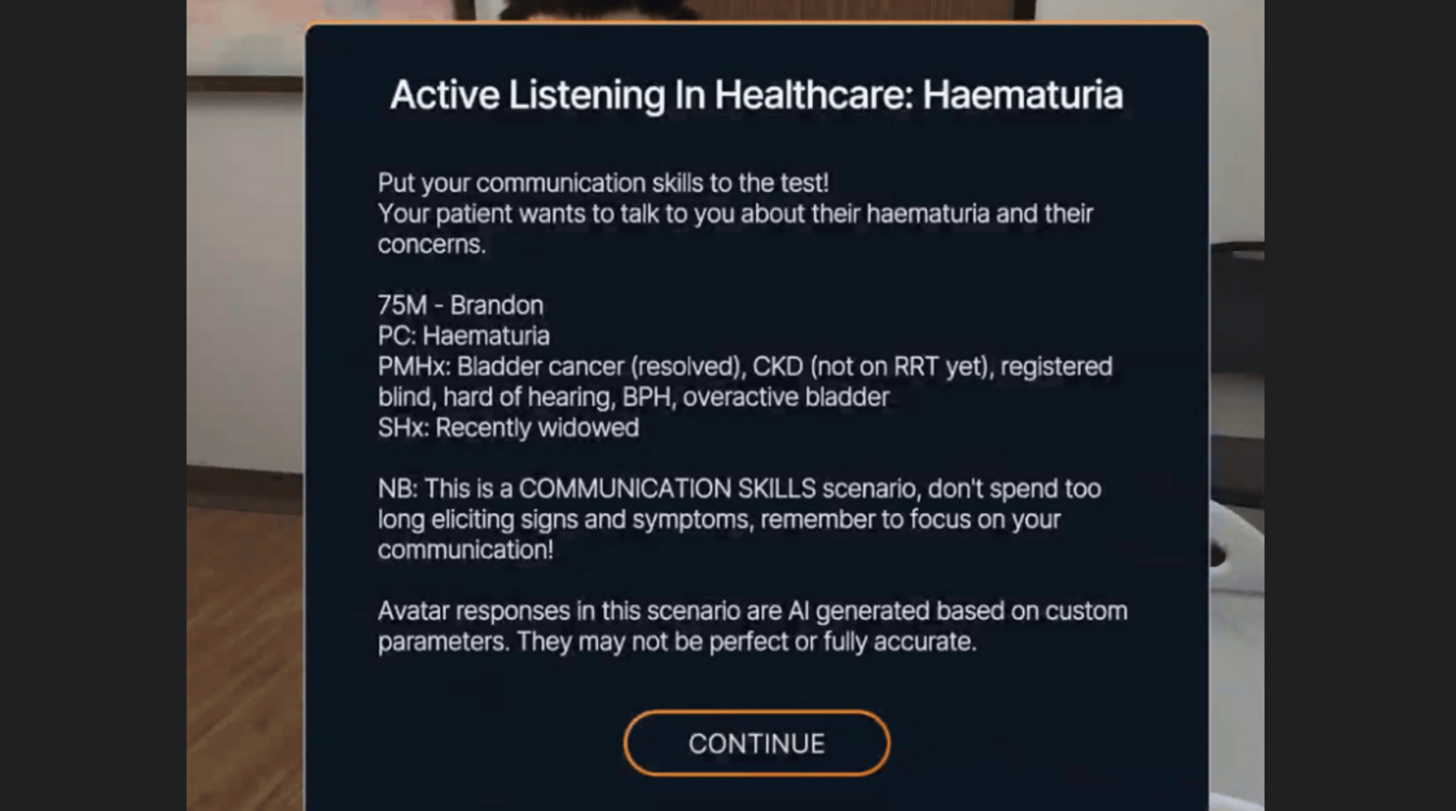
The avatar profile

We developed an AI-enhanced virtual reality scenario simulating haematuria using Bodyswaps© software (Bodyswaps Ltd., London, United Kingdom) and the Meta 1 VR headset (Meta Platforms, Inc., Menlo Park, California). Avatar profiles, including age, presenting complaint, past medical history, and social background, were input into the system (Figure 1).

**Figure 2:**
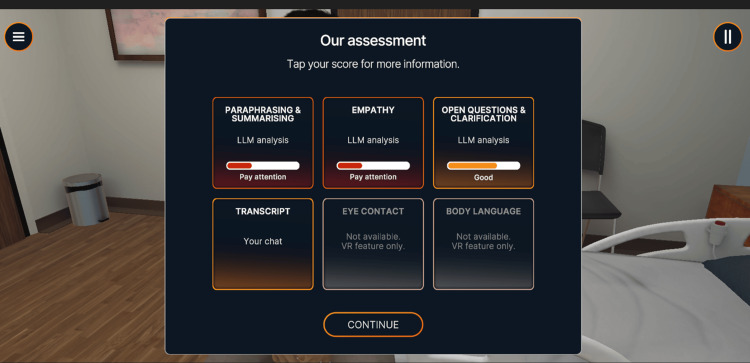
Post-simulation assessment

Drawing on these data, the AI avatar engages in realistic dialogue with participants, mimicking clinical interactions and nuanced communication patterns. The software system provided immediate feedback to each candidate after completion of the scenario (Figure 2).

Recruitment

Between May and July 2025, we recruited a total of 46 clinical professionals at Newham University Hospital (NHS) for participation in the study, with no exclusion criteria applied.

Study design

**Figure 3:**
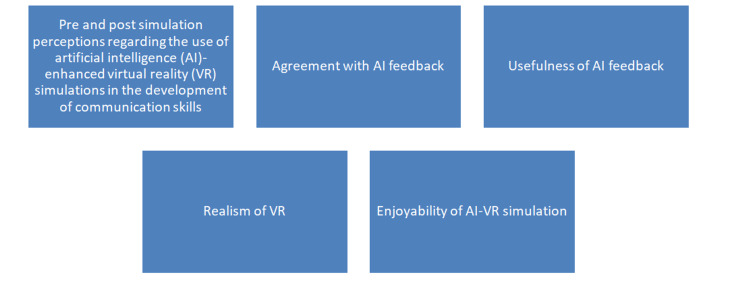
The questionnaire components

This study employed a single-group design to evaluate healthcare professionals' perceptions of an AI-enhanced virtual reality (VR) tool for teaching and assessing urology-related communication skills. All participants received standardised instruction on using the VR headset and Bodyswaps© software before engaging with the haematuria scenario. Participants anonymously completed pre- and post-scenario questionnaires using a five-point scale (1 = strongly disagree, 3 = neutral, 5 = strongly agree) (Figure 3). No control group was included, as the study relied on the participants’ baseline clinical experience for comparative evaluation.

Ethics

Participation in this study was completely voluntary, with informed verbal consent obtained from all participants, and all data were anonymous. A formal NHS Research Ethics Committee review was not required according to the Medical Research Council and NHS Health Research Authority online tool [14].

Data analysis

We performed one-sample t-tests against a neutral midpoint (3.0) using GraphPad Prism 10 AI-powered software (GraphPad Software, LLC, San Diego, California).

## Results

**Figure 4:**
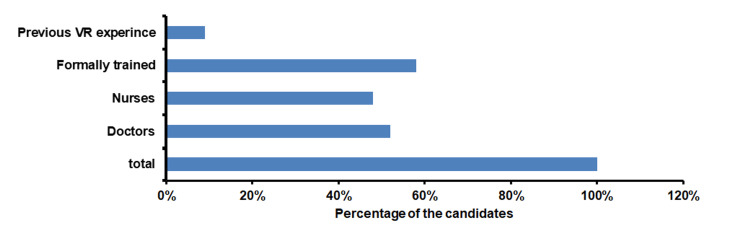
Participants and their previous VR exposure VR: virtual reality.

A total of 45 healthcare professionals (N = 45) participated in the study, including physicians (n = 23, 52%) and nurses (n = 22, 48%). Almost half of the participants (n = 26, 58%) reported having received formal communication skills training at some point during their clinical careers, and the majority of participants (n = 41, 91%) indicated that they had never previously used AI-enhanced VR technology to practise communication skills (Figure 4).

**Figure 5:**
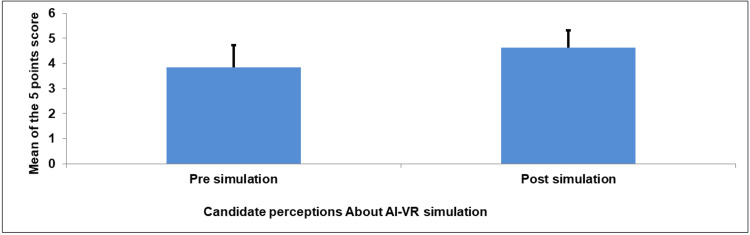
Pre- and post-simulation perceptions regarding the use of artificial intelligence (AI)-enhanced virtual reality (VR) simulations in the development of communication skills

The analysis revealed statistically significant positive perceptions regarding the use of AI-enhanced VR simulations in the development of communication skills among healthcare trainees. Prior to the simulation experience, participants (n = 23, 51.11%) expressed a moderate level of agreement regarding the potential of AI-VR to improve communication skills, with approximately half responding above the neutral midpoint (mean (M) = 3.844 ± 0.9034, p = 0.0001). Following the simulation, participants (n = 40, 88.89%) perceived the AI-powered simulation as useful, showing a substantial increase, with the majority responding above the neutral midpoint (M = 4.636 ± 0.6851, p = 0.0001) (Figure 5).

**Figure 6:**
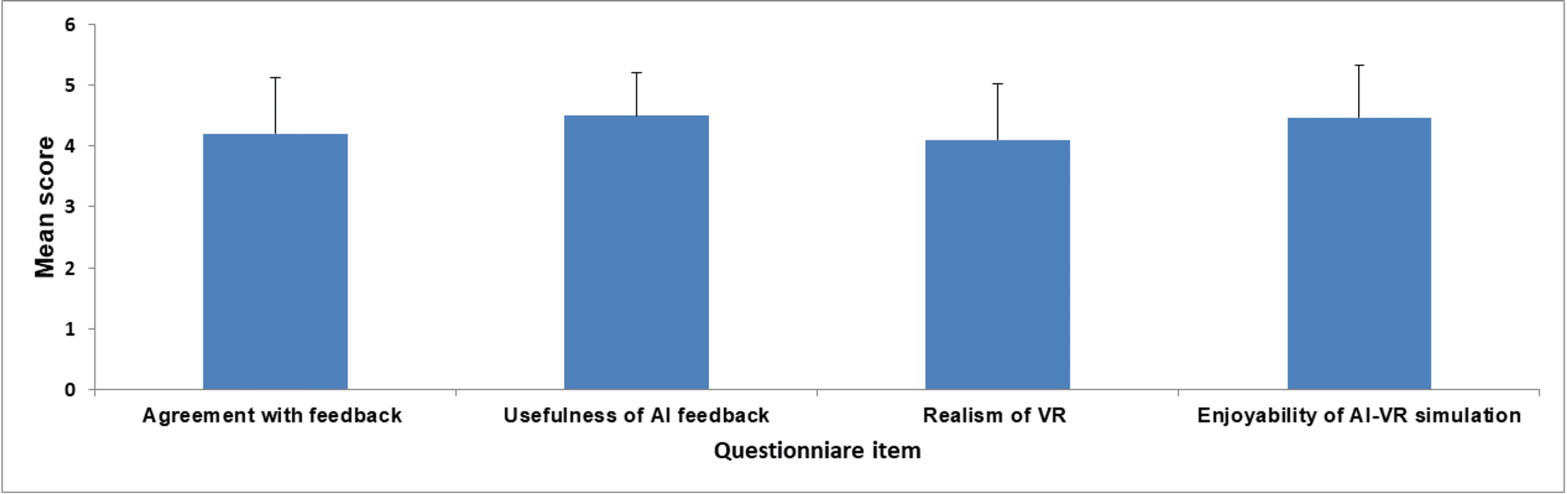
Participants evaluations and statistical analysis of the AI-enhanced VR simulation

Participants (n = 35, 77.78%) also expressed agreement with the feedback generated by the AI system, with high responses above the neutral midpoint (M = 4.2 ± 0.9195, p < 0.0001). Additionally, the AI-generated feedback was rated as highly useful by participants (n = 40, 88.89%) responding above the neutral midpoint (M = 4.5 ± 0.6949, p < 0.0001). The immersive quality of the simulation was positively rated by participants (n = 34, 73.33%), acknowledging the realism of the VR experience in comparison to actual clinical practice (M = 4.159 ± 0.9135; p < 0.0001). Finally, the AI-VR simulation was rated as enjoyable by healthcare professionals (n = 41, 91.11%), with the majority responding above the neutral midpoint (M = 4.465 ± 0.8549, p < 0.0001) (Table 1, Figure 6).

**Table 1:** Participants ratings and statistical analysis of AI-enhanced VR simulation

Item Description	N	Mean (M)	SD	T, df	P-value	Significant (Alpha = 0.05)
Agreement with AI feedback	45	4.2	0.9195	t = 30, df = 44	<0.0001	yes
Usefulness of AI feedback	45	4.5	0.6949	t = 43, df = 44	<0.0001	yes
Realism of VR	44	4.1	0.9135	t = 30, df = 43		
Enjoyability of AI-VR simulation	43	4.465	0.8549	t = 34, df = 42	<0.0001	yes

Across all evaluated items, the p-values were below the threshold of 0.05, indicating statistically positive perceptions of the AI-enhanced VR simulation. These results support the integration of such technology into communication training in healthcare education.

The majority of participants provided additional comments post-simulation, and a few examples are presented below:

“More interesting”, “It provides an enclosed environment to practise and garner skills to equip you for the medical workplace”, “Was surprised at how good the AI patient was and how close to real life the interaction felt”, “Surprised how real it is”, “IT and internet connection to improve”, “IT issues, connection with voice”, and “Interesting usage of AI, could be a supplemental tool but should not replace interaction with patients or actors”.

## Discussion

The results of this pilot study highlight the positive reception of AI-enhanced VR simulations for developing communication skills in urology. The study sample included nearly equal numbers of physicians and nurses. While more than half of the participants had received formal communication skills training at some point in their careers, the majority had never utilised AI-powered VR technology in their clinical training, making their feedback particularly valuable.

Participants showed a statistically significant improvement in their perception of the usefulness of AI-VR technology for enhancing communication skills following the simulation, with an increase from pre-simulation evaluations. These results are consistent with previous research indicating that immersive technologies can substantially enhance learner engagement and perceived skill development in medical education settings [8,15,16].

The agreement with and perceived usefulness of AI-generated feedback demonstrate the potential of AI to provide personalised and real-time assessments that facilitate reflective learning, which is an essential element in the development of professional competency [17]. Additionally, participants rated the realism of the simulation positively, highlighting the ability of AI-enhanced VR to replicate complex clinical environments and interactions, which could be challenging to simulate in traditional educational settings [18,19].

The high enjoyability of the simulation further suggests that such platforms can provide sustained engagement, which is an important factor for long-term educational retention [20]. Participants also reported that the simulation was both engaging and beneficial, with comments such as “It provides an enclosed environment to practise and develop skills for the medical workplace” and “I was surprised at how good the AI patient was and how close to real-life the interaction felt.” These comments reflect the engaging and realistic nature of the experience, which may have contributed to the high ratings for both the simulation's usefulness and enjoyment.

Some technical challenges were noted, particularly related to IT issues and voice connection problems, which may have slightly detracted from the overall experience. These issues highlight the need for further refinement in the integration of AI and VR technologies into healthcare training platforms. Taken together, the results of this pilot study contribute to the growing body of evidence supporting the integration of AI-powered VR platforms as effective tools for communication training in health professions education.

This study has several limitations that should be acknowledged. First, the small number of participants, who were volunteers, may introduce selection bias, as those with a pre-existing interest in technology or communication training may have been more likely to participate. Second, the absence of a control group receiving conventional human-based communication training limits the ability to directly compare the effectiveness of the AI-enhanced VR simulation against standard educational methods. Lastly, the questionnaire was not validated, and the study did not evaluate cost-effectiveness.

## Conclusions

The results of this study support that AI-enhanced VR simulations are perceived by healthcare professionals as engaging tools for developing communication skills in clinical education, particularly as exposure to the AI-powered simulation increased participants' recognition of its educational value. The high ratings for the usefulness of the simulation, agreement with AI-generated feedback, perceived realism, and overall enjoyment demonstrate the potential of interactive technologies to replicate complex clinical scenarios and encourage learner engagement. Future research should incorporate a control group to compare AI-VR with traditional human-based communication training, include larger and more diverse sample sizes to enhance generalisability, and evaluate long-term outcomes such as knowledge retention and the application of skills in real-world settings. Additionally, further exploration of adaptive AI feedback and multi-user VR environments could improve the learning experience. Finally, assessing the cost-effectiveness of AI-VR platforms will be essential for informing their implementation in healthcare education.
